# The Association of Loneliness with Diabetes Is Mediated by Physical Activity and BMI but Not Diet Quality

**DOI:** 10.3390/nu15234923

**Published:** 2023-11-25

**Authors:** Marie Fanelli Kuczmarski, Elizabeth Orsega-Smith, Michele K. Evans, Alan B. Zonderman

**Affiliations:** 1 Laboratory of Epidemiology and Population Sciences, National Institute on Aging, National Institutes of Health, Baltimore, MD 21224, USA; evansm@grc.nia.nih.gov (M.K.E.);; 2 Department of Health Behavior and Nutrition Sciences, University of Delaware, Newark, DE 19716, USA; eosmith@udel.edu

**Keywords:** diet quality, physical activity, loneliness, diabetes

## Abstract

Loneliness is considered a predictor of poor health through numerous pathways. Mediators of this association has not been extensively explored. The study objective was to determine if diet quality and physical activity are parallel mediators with body mass index (BMI) as the third mediator in the association of loneliness with diabetes. The sample, middle-aged and older African American and White adults, 36–77 years, participated in the second follow-up wave of the prospective Healthy Aging in Neighborhoods of Diversity across the Life Span study, 2013–2017. Loneliness was measured by the UCLA 3-item loneliness scale. Participants were categorized as not diabetic, pre-diabetic, or diabetic based on fasting blood glucose, self-reports, or taking medication for diabetes. The Mean Healthy Eating Index-2010 score was calculated from two 24 h dietary recalls collected using the USDA automated multiple pass method. Physical activity was derived from the Baecke questionnaire. The Hayes PROCESS macro, model #80, was used to perform the mediational analysis. Covariates were age, sex at birth, race, income, alcohol intake, and education. Loneliness was inversely and significantly associated with diet quality and physical activity. The only significant indirect path was loneliness > physical activity > BMI > diabetes. Better understanding of modifiable lifestyle behaviors when developing interventions may improve mental health, thereby improving health.

## 1. Introduction

Loneliness results from a perceived discrepancy between desired and achieved levels of social contact and is distinct from social isolation, which is an objective construct [1]. Loneliness, a subjective, unpleasant emotional state, is oftentimes associated with increased risk for diabetes and cardiovascular disease [2,3,4], making it a serious public health concern. There are other strong predictive relationships between loneliness and health conditions such as increased depression, impaired cognitive performance, and, with age, increased likelihood of nursing home admission [5,6]. Loneliness may also directly affect lifestyle behaviors [7,8,9,10,11]. For instance, data from the English Longitudinal Study of Ageing revealed loneliness was negatively associated with successful smoking cessation [12].

Several explanatory factors representing different pathways, namely behavioral, biological, and psychological, may contribute to the association of loneliness with negative health outcomes [1,13]. Loneliness is thought to influence health through biological mechanisms, as is sometimes evidenced by independent associations with increased inflammatory and stress biomarkers [14,15,16,17]. Dietary practices, physical activity, intake of alcoholic beverages, smoking, and sleep patterns are among the behavioral factors that have been explored to partially explain the association [18]. Psychological factors, such as perceived stress and depression, have been explored with inconsistent effects on the association [19]. Sociodemographic factors, potential confounders, can also affect the association of loneliness with poor health [8,20]. 

The challenge in exploring the effects of loneliness is its multifaceted nature and its complex associations to health, including both intrinsic (e.g., medical conditions and genetics) and extrinsic (e.g., social and physical environment) factors [13]. There is evidence that loneliness is a strong predictor of diabetes [2]. A review of the nutrition literature provides evidence of the scarcity of research on the effects of diet quality on the association between loneliness and diabetes. It is recognized that diet quality is associated with increased risk for pre-diabetes [21], and that among persons with diabetes, the lowest quartiles of diet quality were associated with higher odds of hyperglycemia and overweight/obesity compared to higher quartiles of diet quality [22].

Much of our understanding about dietary practices and activity has come from examining social isolation, not loneliness [23,24]. Data from the SHARE (Survey of Health, Ageing and Retirement in Europe) project found that, in 9 European countries, highly socially isolated individuals were more likely to be physically inactive and, in 14 European countries, high social isolation increased the likelihood of having an inadequate diet, especially fruit and vegetable consumption [25]. Jiang and colleagues found loneliness was associated with both unhealthy dietary behaviors, reflected in low Healthy Eating Index (HEI) scores, and physical inactivity among college students [7]. Loneliness was found to be associated with poor dietary habits and lower adherence to the Mediterranean diet in adolescents, in comparison to their counterparts with low perceived loneliness [9]. In adults aged 60 to 94 years, loneliness was associated with nutrient inadequacies [24].

The association of loneliness with diabetes should also consider body weight. It is widely recognized that obesity is a risk factor for type 2 diabetes [26,27]. There is evidence of the association between loneliness and obesity [28,29,30]. However, it is unclear if obesity is the sequel to loneliness due to inconsistency in findings among studies. Like loneliness, obesity is complex and involves behavioral, environmental, sociocultural, and psychological factors [31]. Sedentary lifestyles, long-term energy imbalance, and unhealthy diets are included among the list of contributors to overweight and obesity. Jung and Luck-Sikorski recommended future studies investigate the mediation pathways between obesity, loneliness, and its determinants to provide a framework for successful health interventions [29].

Our knowledge of the potential differences in the indirect behavioral pathways of loneliness to diabetes is limited. The maintenance of quality of life and physical and mental well-being in later life is dependent on healthy behaviors. Better understanding of lifestyle factors that could reduce risk of poor health in lonely individuals warrants further investigation. The objective of this study is to determine if diet quality, physical activity, and body mass index (BMI) mediate the association of loneliness with diabetes in middle-aged and older African American and White adults. We hypothesized that loneliness affects diet quality and engagement in physical activity and that these lifestyle behaviors influence BMI, mediating the association between loneliness and diabetes. 

## 2. Materials and Methods

### 2.1. Sample

Individuals in this study participated in the second follow-up visit in the Healthy Aging in Neighborhoods of Diversity across the Life Span (HANDLS) study, 2013–2017. The design of HANDLS study was a 4-way factorial of age, sex, race, and income. Details of this study are published elsewhere [32]. Briefly, the prospective cohort in HANDLS study was initiated in 2004 to examine the influence of race and socioeconomic factors in health disparities in African American and White adults residing in 13 predetermined neighborhoods in the urban United States (U.S.) city of Baltimore. The baseline cohort consisted of 3720 persons, of which 2147 participated in the second follow-up visit. The present analytic sample consisted of participants with complete data on predictor, outcome, and explanatory variables (*n* = 1713).

The study protocol was approved by Human Institutional Review Board at the National Institutes of Health. Each participant provided written informed consent and was financially compensated.

### 2.2. Predictor Variable: Loneliness

Loneliness was measured by the UCLA three-item loneliness scale [33]. The three questions were: How often do you feel that you lack companionship?; How often do you feel left out?; and How often do you feel isolated from others? The response categories were coded 1 (hardly ever), 2 (some of the time), and 3 (often). Each participant’s responses to the three questions were summed, with higher scores indicating greater loneliness. The possible maximum score was 9. The three-item loneliness scale has been shown to have satisfactory reliability and both concurrent and discriminant validity [33]. 

### 2.3. Behavioral Lifestyle Factors

#### 2.3.1. Diet

Each participant completed two 24 h recalls collected by trained interveners using automated multiple pass method (AMPM) created by the U.S. Department of Agriculture (USDA) [34]. The first recall was obtained in-person and the second by telephone. A food model booklet and other measurement aids were used to assist participants with portion size estimation. All foods and beverages reported were assigned food codes using the USDA Food and Nutrient Database for Dietary Studies 2013–2014. 

Diet quality was assessed by the Healthy Eating Index (HEI)-2010, an index that measures adherence to recommendations from the Dietary Guidelines for Americans [35]. A detailed description of the procedure used to calculate the HEI-2010 is available on the HANDLS website [36]. The basic steps to calculate the HEI-2010 component and total scores and the statistical codes for 24 h dietary recalls were provided on the National Cancer Institute’s Applied Research website [37]. Component and total HEI-2010 scores were calculated for each recall day and were averaged to obtain the mean for both days combined. The maximum possible score was 100.

#### 2.3.2. Physical Activity

For this study, two domains of the validated Baecke questionnaire [38] were used to obtain information on physical activity. The Baecke physical activity questions were incorporated into the audio computer-assisted self-interview assessment [32]. The HANDLS study participants were asked to report if they engaged in “sports” and “non-sports leisure” activities, the intensity of sport activities (moderate and vigorous activity), and the time spent in sports and leisure activities. The time (minutes/week) spent in moderate, vigorous, and leisure activities was summed for each participant. There is evidence that the Baecke questionnaire has good validity in community-dwelling adults when compared to accelerometry, with better accuracy and sensitivity among participants with medium-to-high education levels [39].

The “sports” component of the physical activity criteria included the following self-reported questions:

- Do you play sports or are you physically active in your leisure time or time awake? (yes/no)
- What sport or physical activity do you do most frequently? (low intensity e.g., walking, moderate intensity, e.g., biking, high intensity, e.g., basketball)
- How many hours a week do you play or do your most frequent activity? (<1 h, 1–2 h, 2–3 h, 3–4 h, >4 h)
- What sport or physical activity do you do next most frequently? (low intensity e.g., walking, moderate intensity, e.g., biking, high intensity, e.g., basketball)

The “non-sports leisure” domain included the following self-reported questions:

- During leisure time, I watch television (five-point Likert scale from never, seldom, sometimes, often, to very often)
- During leisure time, I walk (five-point Likert scale from never, seldom, sometimes, often, to very often)
- During leisure time, I cycle (five-point Likert scale from never, seldom, sometimes, often, to very often)
- How many minutes per day do you walk or cycle to and from work or shopping? (<5 min, 5–15 min, 15–30 min, 30–45 min, >45 min)

For “sports”, specific questions on hours per week and months per year of participation were asked, while for “leisure”, time in minutes per day was asked. To calculate minutes per day of moderate and vigorous sports activity and leisure activity, the median time of the range was used:

- For sport activities: <1 h ~ 30 min; 1–2 h ~90 min; 2–3 h ~150 minutes.; 3–4 h ~210 min; >4 h ~270 min.
- For non-sports leisure activities: (<5 min ~2.5 min; 5–15 min ~10 minutes; 15–30 min ~22.5 min; 30–45 min ~37.5 min; >45 min ~52.5 min.

#### 2.3.3. Body Mass Index

BMI was calculated from measured weight and height as a ratio of weight to height squared, kg/m^2^. A calibrated Med-weigh, model 2500, digital scale was used to measure weight. Height was measured with the HANDLS study participant’s heels and back against a height meter (Novel Products, Inc., Rockton, IL, USA). 

### 2.4. Covariates

Race was self-reported only at the baseline visit as African American or White. Similarly, sex at birth was only reported at baseline and coded as male or female. Participants were categorized as above or below poverty status defined by 125% of the 2004 U.S. Health and Human Services Poverty Guidelines, also only at baseline enrollment [40]. Age at the second follow-up visit was coded in years. Mean alcohol intake in grams was calculated based on the 24 h recalls. Education was self-reported and coded as years completed.

### 2.5. Outcome Variable: Diabetes

Diabetes was coded as not diabetic, pre-diabetic, or diabetic based on three measures, namely fasting glucose levels, self-reports, and taking medication for diabetes. For the mediation analysis, this variable was recoded as a dichotomous variable; not diabetic or pre-diabetic/diabetic. Among people with diabetes in the HANDLS study, it was not possible to distinguish those with type 1 from type 2 in our analytical sample.

### 2.6. Statistical Analysis

Means and standard errors for continuous variables and the proportion of participants for relevant categorical variables were calculated. Analysis of variance (ANOVA) was used to compare demographic and lifestyle factors and loneliness scores between races (African American and White). For sample characteristic categorical data, χ^2^ tests were used. Statistical significance was established at *p* < 0.05. All statistical analyses were performed with IBM SPSS Statistics for Windows Version 29 (2022; IBM Corporation, Armonk, NY, USA).

**Figure 1:**
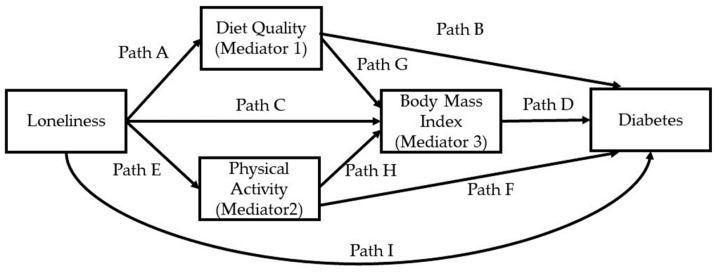
Conceptual model for exploring mediators in the association of loneliness with diabetes, adjusting for covariates. Paths A–I in model represent direct effects. Diet quality and physical activity are parallel mediators, which are serially antecedent to body mass index, which is the third mediator.

To examine the mediating roles of diet quality, physical activity, and BMI on the association of loneliness with diabetes, the PROCESS macro, model #80, for SPSS Version 4.2 by A Hayes was used [41]. PROCESS is an observed variable ordinary least-squares and logistic regression path analysis modeling tool. A dichotomous outcome variable can be used with the macro. The conceptual model for the mediation analysis is displayed in Figure 1. Ordinary least-squares regression is the estimation approach utilized in the PROCESS macro to compute the indirect effect. Model #80 of the PROCESS macro is comprised of 4 submodels. Models 1 and 2 involve regressing each of the parallel mediators, namely diet quality and physical activity, onto loneliness using simple linear regression. Paths A and B in Figure 1 show where diet quality and physical activity are regressed onto loneliness, respectively. Model 3 involves regressing BMI simultaneously onto loneliness, diet quality, and physical activity. Model 4 involves regressing diabetes onto loneliness, and the three mediators. The PROCESS macro, model #80, includes 9 direct effects, displayed in Figure 1 as Paths A-I. Five specific indirect effects are computed as the product of path coefficients (Path A × Path B; Path A × Path G × Path D; Path C × Path D; Path E × Path F; Path E × Path H × Path D). The predictor and mediators in our study were continuous variables. The unstandardized b (or beta) coefficients generated from the PROCESS macro was the log odds ratio, Loge (OR). To simplify interpretation of coefficients, the Loge (OR) were converted to odds ratios [42]. 

The indirect effect is unstandardized since the outcome, diabetes, was a dichotomous variable. The default in PROCESS for the indirect effect is bootstrapped 95% confidence intervals. If the lower and upper confidence intervals are greater than zero and the values are positive, the conclusion is the indirect effect and is significant at a *p* < 0.05. 

## 3. Results

### 3.1. Sample Characteristics

Characteristics of the overall analytical sample and the sample stratified by race are provided in Table 1. The mean age (±SE) of the overall sample was 56.6 ± 0.2 years. Approximately 59% of the analytical sample were female, 39.1% White adults, and 37.7% had income below the 125% poverty level. Of the sample, 24% had diabetes and roughly 16% were classified as pre-dietetic. About 14% of the sample reported that they often felt a lack of companionship, but only ~8% indicted they often felt left out or isolated from others (Table 1).

The analytical sample categorized by race did not reveal differences in mean age or sex (Table 1). There were also no differences in the mean HEI-2010 scores, loneliness scores, minutes of weekly physical activity, or BMI by race. Approximately 24% of the analytical sample had a normal weight (BMI ≤ 24.9), 26% were overweight (BMI between 25.0 and 29.9), and 51% were obese (BMI ≥ 30). There were differences by race in the frequency of feeling left out, with a higher percent of White adults responding “often” compared to African American adults (*p* = 0.033). A greater percentage of African American adults had diabetes, while pre-diabetes was more prevalent among White adults (*p* = 0.008) (Table 1). The mean years of education was significantly higher for White adults compared to African American adults, 12.4 vs. 12.2 y, respectively (*p* = 0.042) (Table 1)

### 3.2. Summary of Model #80 of PROCESS Macro

**Figure 2:**
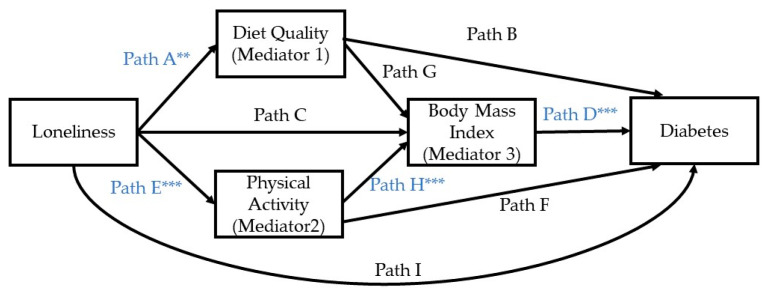
Significant direct effects based on the PROCESS macro, model #80. ** *p* < 0.01, *** *p* < 0.001.

Of the nine direct paths in model #80, four were found to be statistically significant and are presented in blue font in Figure 2. The direct effects, odds ratios, and 95% confidence intervals for each path are presented in Table 2. Of the five indirect effects, only one was found to be statistically significant (Table 3). This indirect path involves both physical activity and BMI. The values of the five indirect effects, along with the bootstrap 95% confidence intervals, are provided in Table 3. The next four sections of the results describe the four submodels of model #80. 

### 3.3. Model 1. Path A: Diet Quality 

The findings from Model 1 of the PROCESS macro revealed loneliness, the predictor, had a significant and negative direct effect on the mediator diet quality with the covariates included in the model (Table 4). The odds of having a low HEI score increased with loneliness (odds ratio = 0.33, Table 2). Being older age, female, having an income >125% poverty or completing more years of education was significantly associated with higher diet quality (*p* < 0.01) (Table 4). For Model 1, the R-square = 0.1097 (*p* < 0.001) indicating loneliness accounted for ~11% of the variance in diet quality.

### 3.4. Model 2: Path E: Physical Activity

Similar to diet quality, loneliness had a significant and negative direct effect on time spent in physical activity, adjusting for the covariates (Table 5). Being younger, male or having completed more years of education was significantly associated with more physical activity (*p* < 0.001). The R-square of Model 2 equaled 0.0547 (*p* < 0.001) indicating loneliness accounted for approximately 5% of the variance in activity. 

### 3.5. Model 3: Paths C, G, H: Diet Quality, Physical Activity, BMI

For Model 3 loneliness, diet quality and physical activity, the predictors, accounted for significant variance in BMI, R-square 0.0846 (*p* < 0.0001). The predictors combined accounted for approximately 8% of the variation in BMI. As shown in Table 6, loneliness (Path C) and diet quality (Path G) were nonsignificant predictors of BMI, whereas physical activity (Path H) was a negative and significant predictor of BMI. Being younger, female, having an income >125% poverty status, and consuming fewer alcoholic beverages were significantly associated with higher BMI (Table 6).

### 3.6. Model 4: Paths B, D, F, I, and Indirect Effects

For Model 4, the last submodel of model #80, the only significant direct path was between BMI and diabetes (Table 2 and Table 7, *p* < 0.001). This result indicated the greater the BMI, the higher the risk for diabetes. The odds ratio was 1.16. Loneliness as a predictor of diabetes was tending towards significance (*p* = 0.0557, Table 7). The indirect effects generated with this model are provided in Table 3. This model was adjusted for all the covariates. The total indirect effect of loneliness on diabetes through the set of mediators was 0.0046 (bootstrap 95% confidence interval: −0.0139, 0.0229). The only statistically significant indirect effect was the effect of loneliness on diabetes via the sequence of the mediators, physical activity, and BMI. The total effect of loneliness on diabetes equaled 0.0651, the sum of the direct effect of loneliness on diabetes (0.0605) and total indirect effect (0.0046). In this model, similar to models 1–3, race was not a significant factor. Poverty status was also not significant. Being older, male, having completed less education, and consuming fewer alcoholic beverages were significantly associated with diabetes. There were no significant interactions between loneliness and any of the mediators with diabetes. 

## 4. Discussion

To our knowledge, the findings are the first to report that the association between loneliness and being either an African American or White adult with pre-diabetes or diabetes was mediated by the sequence of physical activity to BMI. As anticipated, loneliness had a direct inverse significant association with both diet quality and physical activity. Higher scores for loneliness were associated with lower HEI scores and less time spent in leisure and moderate or vigorous physical activities. These results are consistent with other studies using younger samples [7,9]. When examining the combined effects of loneliness, diet quality, and physical activity on BMI, only activity was a significant and negative predictor. In a systematic review of the literature Pels and colleagues found that physical activity and loneliness were generally inversely related and it may be that decreased physical activity can lead to higher BMIs [43]. Researchers have also reported that those individuals who are lonely typically have higher BMIs [29]. However, to our knowledge, there is a gap in the literature addressing the factors of loneliness, diet quality, and physical activity on BMI, revealing the new contribution of this article. Consistent with other findings reported in the literature, BMI was a positive and significant predictor of diabetes [44,45].

Genetics and modifiable lifestyle risk factors are often the underlying cause for the development of most chronic medical and psychiatric diseases. Type 2 diabetes is considered a lifestyle disease, a disease commonly caused by several lifestyle behaviors, such as unhealthy eating, physical inactivity, excessive alcohol, smoking, and exposure to unsafe environments [46,47]. There is evidence that the number and types of lifestyle diseases are rising with the increasing contribution of psychiatric diseases [47,48]. Type 2 diabetes and depression have a common underlying pathology, namely chronic inflammation. Poor mental health is associated with poor health behaviors [49]. Specifically, the consumption of a diet low in fruits and vegetables and high in soft drinks and fast foods, insufficient physical activity, and poor sleep were associated with high odds of mental distress. Individuals with these behaviors had low household incomes (<$70,000) and were not university educated [49]. In our urban sample, loneliness was significantly correlated with depression (r = 0.59, *p* < 0.001). Our previous research found depression, like loneliness, was inversely and significantly associated with low diet quality [50]. It may be that these negative physical health behaviors contribute to negative mental health.

Conversely, positive mental health was found to be associated with greater consumption of fruit and vegetables. Previous research with HANDLS study data found that, among men, changes in food intake to a diet with more anti-inflammatory potential were significantly associated with enhanced mental health with increasing age [51]. However, there is evidence that diet does not mediate the association of positive mental health on cardiometabolic risk [52]. In the same study, physical activity was shown to have an indirect protective effect on the association between positive mental health and cardiometabolic risk [52]. Furthermore, greater socioeconomic status was associated with lower cardiometabolic risk in this sample [52]. 

In addition to diet and physical activity, an important lifestyle factor that may modify mental health is social interactions [46]. While social connections are viewed as beneficial for mental health, the protective relationship varies by sex and among population groups. Better understanding of the relationships between mental health and lifestyle health behaviors is essential to allow the integration of strategies to address both for improving health outcome related to diabetes. 

The effects on health of combinations of poor lifestyle behaviors appear to be greater than their individual effects, suggesting a synergistic relationship between risk factors [49]. Although more challenging, it seems interventions that target multiple behavior changes may have greater potential for better health outcomes than interventions focused on a single behavior change [48,53]. Developing and targeting intervention programs on both mental health and lifestyle behaviors may improve health outcomes.

This study has strengths. First, the findings contribute to the literature as the urban African American and White sample examined are underrepresented in nutrition studies. Another strength is that the HEI was based on two 24 h recalls collected by a validated method. Loneliness was measured using the UCLA 3-item scale, which is a valid and widely used tool. Limitations of the study also exist. Diet and physical activity were self-reported and can be biased as, typically, individuals over-report positive behaviors and under-report negative behaviors. The participants were predominantly sedentary, indicated by right-skewed data, which may yield larger standard errors than symmetrically distributed independent variables. This might explain why we did not have sufficient power to detect an effect. It was not possible to address all the many factors that play a role in diabetes susceptibility because some variables were not available in our data or a factor like genetic risk was beyond the scope of this manuscript. Last, the study is based on cross-sectional data; thus, one cannot assess casual effects, despite the use of the Hayes PROCESS method for analyses. 

## 5. Conclusions

Behavioral lifestyles such as physical activity and diet are well-established factors with respect to diabetes risk in the general population. Furthermore, loneliness is strongly associated with less physical activity and less than optimal diet quality, which may contribute to its role in developing diabetes in populations. Like other chronic conditions, diabetes is a chronic disease involving inflammation [54]; in contrast, physical activity and a healthy diet may reduce inflammation. Since loneliness is complex, with multiple pathways associated with health [13], further research is warranted to study the multiple possible interactions.

## Figures and Tables

**Table 1:** Characteristics of the HANDLS sample, overall and by race.

Characteristic	Overall Sample *n* = 1713	African American *n* = 1044	White *n* = 669	*p*
Age, y X ± SE	56.6 ± 0.2	56.7 ± 0.3	56.4 ± 0.3	0.416
Male, %	41.0	41.7	40.1	0.510
African American, %	60.9	-	-	-
Income, % <125% poverty	37.7	41.1	32.4	<0.001
Education, y X ± SE	12.3 ± 0.1	12.2 ± 0.1	12.4 ± 0.1	0.042
Alcohol intake, g X ± SE	7.5 ± 0.6	7.7 ± 0.7	7.4 ± 0.9	0.763
Body Mass Index, kg/m^2^ X ± SE	31.0 ± 7.9	31.0 ± 0.2	30.9 ± 0.3	0.753
*Diabetes*				0.008
Diabetic, %	24.0	26.1	20.8	
Pre-diabetic, %	15.9	14.2	18.5	
Healthy Eating Index-2012, X ± SE	49.05 ± 0.29	48.91 ± 0.35	49.26 ± 0.51	0.563
Physical activity, mins/week X ± SE	187 ± 6	190 ± 7	182 ± 9	0.501
*Loneliness:*				
Total score, X ± SE	4.73 ± 0.04	4.68 ± 0.05	4.81 ± 0.07	0.124
*You feel that you lack companionship*				0.394
Often, %	14.4	14.1	14.9	
Some of the time, %	41.0	42.3	39.0	
*You feel left out*				0.033
Often, %	7.5	6.2	9.4	
Some of the time, %	39.1	38.8	39.6	
*You feel isolated from others*				0.067
Often, %	7.8	7.2	8.8	
Some of the time, %	33.3	31.8	35.7	

Abbreviations: kg/m^2^—kilograms per meter-squared; X ± SE—Mean ± Standard Error; y—years.

**Table 2:** Mediation analysis summary of direct effects based on Hayes PROCESS macro, model #80 ^a^.

Paths	Direct Effect ^b^	*p*	Odds Ratio	Confidence Interval	
				Lower Bound	Upper Bound
A: Loneliness -> Diet quality	−0.485 ± 0.166	**0.004**	0.33	0.15	0.69
B: Diet Quality -> Diabetes	0.006 ± 0.005	0.214	1.01	0.99	1.03
C: Loneliness -> Body mass index	−0.021 ± 0.112	0.852	0.95	0.58	1.58
D: BMI -> Diabetes	0.065 ± 0.007	**<0.001**	1.16	1.12	1.20
E: Loneliness -> Physical activity	−16.702 ± 3.305	**<0.001**	1.98 × 10^−17^	6.54 × 10^−24^	6.04 × 10^−11^
F: Physical activity -> Diabetes	−0.0002 ± 0.0002	0.382	1.00	1.00	1.00
G: Diet quality -> BMI	0.030 ± 0.016	0.068	1.07	0.99	1.15
H: Physical activity -> BMI	−0.006 ± 0.001	**<0.001**	0.99	0.98	0.99
I: Loneliness -> Diabetes	0.061 ± 0.032	0.056	1.15	1.00	1.33

^a^ The PROCESS model adjusted for age, race, sex, poverty status, alcohol intake, and years of education. ^b^ Beta coefficients±Standard Error; Coefficients are log odds ratios [Loge (OR)]. Abbreviation: OR—odds ratio.

**Table 3:** Mediation analysis summary of indirect effects based on Hayes PROCESS macro, model #80 ^a^.

Relationship	Indirect Effect ^a^	Bootstrap Confidence Interval	
		Lower Bound	Upper Bound
Loneliness -> Diet quality -> Diabetes	−0.0028 ± 0.0025	−0.0086	0.0013
Loneliness -> Physical activity -> Diabetes	0.0035 ± 0.0041	−0.0042	0.0123
Loneliness -> Body mass index -> Diabetes	−0.0014 ± 0.0080	−0.0172	0.0139
Loneliness -> Diet quality -> BMI ->Diabetes	−0.0009 ± 0.0006	−0.0024	0.0000
Loneliness -> Physical activity -> BMI -> Diabetes	0.0061 ± 0.0015 *	0.0036	0.0094

^a^ PROCESS model was adjusted for age, race, sex, poverty status, alcohol intake, and years of education. * *p* < 0.05 Abbreviation: OR—odds ratio.

**Table 4:** Model 1 output with Healthy Eating Index-2010 as outcome.

Outcome: Heathy Eating Index	Coefficient Loge (OR)	Standard Error	t	*p*	Confidence Intervals	
					Lower Bound	Upper Bound
Constant	36.2309	2.9483	12.2888	0.0000	30.4483	42.0136
Loneliness	−0.4845	0.1663	−2.9131	0.0036	−0.8107	−0.1583
Age	0.1244	0.0309	4.0265	0.0001	0.0638	0.1850
Race	0.0372	0.5691	0.0653	0.9479	−1.0791	1.1534
Sex	−3.0711	0.5651	−5.4342	0.0000	−4.1796	−1.9627
Poverty	−1.6233	0.5910	−2.7467	0.0061	−2.7825	−0.4642
Alcohol intake	0.0271	0.0121	2.2459	0.0248	0.0034	0.0508
Education	1.1700	0.1127	10.3835	0.0000	0.9490	1.3910

**Table 5:** Model 2 output with physical activity as outcome.

Outcome: Physical Activity	Coefficient Loge (OR)	Standard Error	t	*p*	Confidence Intervals	
					Lower Bound	Upper Bound
Constant	315.5284	58.5894	5.3854	0.0000	200.6138	430.4430
Loneliness	−16.7017	3.3051	−5.0533	0.0000	−23.1842	10.2192
Age	−3.8177	0.6140	−6.2183	0.0000	−5.0219	−2.6135
Race	9.1480	11.3098	0.8089	0.4187	−13.0345	31.3305
Sex	44.0386	11.2308	3.9212	0.0001	22.0109	66.0662
Poverty	−8.5966	11.7445	−0.7320	0.4643	−31.6318	14.4386
Alcohol intake	0.1375	0.2398	0.5734	0.5664	−0.3328	0.6078
Education	8.1480	2.2393	3.6387	0.0003	3.7560	12.5399

**Table 6:** Model 3 output with body mass index as outcome.

Outcome: BMI	Coefficient Loge (OR)	Standard Error	t	*p*	Confidence Intervals	
					Lower Bound	Upper Bound
Constant	41.0491	2.0533	19.9922	0.0000	37.0219	45.0762
Loneliness	−0.0208	0.1116	−0.1866	0.8520	−0.2396	0.1980
HEI	0.0296	0.0162	1.8248	0.0682	−0.0022	0.0614
Physical activity	−0.0056	0.0008	−6.8672	0.0000	−0.0072	−0.0040
Age	−0.0747	0.0209	−3.5704	0.0004	−0.1157	−0.0336
Race	0.3106	0.3784	0.8208	0.4119	−0.4316	1.0529
Sex	−2.8089	0.3813	−7.3665	0.0000	−3.3568	−2.0610
Poverty	−1.0160	0.3938	−2.5799	0.0100	−1.7883	−0.2436
Alcohol intake	−0.0384	0.0080	−4.7818	0.0000	−0.0542	−0.0227
Education	−0.0799	0.0774	−1.0329	0.3018	−0.2316	0.0718

**Table 7:** Model 4 output with diabetes as the outcome.

Outcome: Diabetes	Coefficient Loge (OR)	Standard Error	t	*p*	Confidence Intervals	
					Lower Bound	Upper Bound
Constant	−5.1235	0.6699	−7.6477	0.0000	−6.4365	−3.8104
Loneliness	0.0605	0.0316	1.9136	0.0557	−0.0015	0.1225
HEI	0.0057	0.0046	1.2426	0.2104	−0.0033	0.0147
Physical activity	−0.0002	0.0002	−0.8747	0.3817	−0.0007	0.0003
BMI	0.0651	0.0072	8.9850	0.0000	0.0509	0.0793
Age	0.0450	0.0061	7.3665	0.0000	0.0330	0.0569
Race	0.0246	0.1074	0.2288	0.8190	−0.1859	0.2351
Sex	0.4198	0.1104	3.8034	0.0001	0.2035	0.6361
Poverty	−0.0974	0.1122	−0.8685	0.3851	−0.3173	0.1224
Alcohol intake	−0.0057	0.0028	−2.0452	0.0408	−0.0112	−0.0002
Education	−0.0712	0.0221	−3.2216	0.0013	−0.1146	−0.0279

## Data Availability

Data are available upon request to researchers with valid proposals who agree to the confidentiality agreement as required by our Institutional Review Board. We publicize our policies on our website https://handls.nih.gov (accessed on 10 November 2023). Requests for data access may be sent to Alan Zonderman (co-author) or the study manager, Jennifer Norbeck, at norbeckje@mail.nih.gov.
